# Supplementary material for: Experimental quantum compressed sensing for a seven-qubit system
Source: Nat Commun. 2017 May 17;8:15305. doi: 10.1038/ncomms15305 (PMC5442320; doi:10.1038/ncomms15305)
Supplement: Supplementary Information — Supplementary figures, supplementary discussion and supplementary references. [file ncomms15305-s1.pdf]

Supplementary discussion: Experimental quantum compressed sensing for a seven-qubit system

ADDITIONAL EXPERIMENTAL RESULTS

In this supplement, for completeness, we show the results for the 2 additional states that were prepared in the laboratory. In Supplementary Figures 1 and 2 we compare the reconstructed states for the anticipated $|\bar{1}\rangle$ and $(|\bar{0}\rangle + |\bar{1}\rangle)/\sqrt{2}$ encoded state vectors, respectively. The results are qualitatively and quantitatively similar to those in Fig. 1 of the main text. As before, we can see how the `TNM` with minimal ϵ delivers estimates that are comparable with the `LS` and spectral thresholding estimators.

In addition, in Supplementary Figure 3, we show how the `TNM` with high ϵ value leads to close-to-pure (low-rank) estimates for the three states used in this work: $|\bar{0}\rangle$, $|\bar{1}\rangle$ and $(|\bar{0}\rangle + |\bar{1}\rangle)/\sqrt{2}$. As argued in the main text, operating the `TNM` estimator in this regime could be used to determine coherent errors. Also note that, since the anticipated states, which we use to compute the fidelity, are pure states, we observe very high fidelity values in this regime.

SIMULATIONS ON SPECTRAL THRESHOLDING

In this supplement, we test our model selection protocol (eq. (16) of the main text) by performing numerical simulations in a relatively small system of $L = 4$ qubits. We proceed by generating random states of fixed rank: in this example, we choose ranks 1, 2, 4, and 8. These states, which we refer to as the true states ρ, are used to simulate the outcomes of a set of 1, 10, 16, 32, 56, and 81 Pauli basis measurements (measurement settings). Each simulated measurement, in turn, is done for different repetitions per observable. We choose, for comparison, 5, 16, and 100 repetitions per measurement setting. More repetitions means less noise in the observed outcomes. Each numerical experiment is repeated 100 times and the reconstruction of the density matrix is done via the least squares estimator `LS`. Then, we compute our figure of merit M_j, as defined in the main text.

The results are presented in Supplementary Figures 4-7. It is clear from the plots that our method—when given enough measurements—can, in principle, distinguish the correct rank of the true state (see Supplementary Figure 8). However, in the informationally incomplete regime (low data) that we are interested in, it tends to give a lower rank than the true rank. This is indeed a desirable feature since the amount of collected data is not enough to justify a higher rank fit.

Additionally, for benchmarking, we compute the expected risk defined by

$$\mathbb{E}\|\rho - \rho_k\|_2^2, \tag{1}$$

where ρ is the true state and ρ_k is the reconstructed and truncated state from spectral thresholding. This is shown in Supplementary Figure 10.

Since the procedure outlined in this work is related to model selection, for completeness, we compare our method to the one proposed in Supplementary Reference 1. There, the authors have developed a particular spectral thresholding algorithm that is applied to a reconstructed density matrix via a plain least squares estimator that does not impose the positivity constraint. Furthermore, their approach is valid for informationally complete measurements and rigorous proofs are given for its performance. In our test, we have modified the approach in Supplementary Reference 1 to include the positivity constraint (via the `LS` estimator) and have naively applied it to the regime of informationally incomplete measurements. As suggested in Supplementary Reference 1, we choose the threshold parameter as

$$\gamma(\varepsilon)^2 = \frac{2d}{N}\log\frac{2d}{\varepsilon}. \tag{2}$$

In our example, we select $\varepsilon = 0.05$ and set N to be the total number of prepared quantum states used in the simulation of a particular experiment. For every reconstructed density matrix, computed via our `LS` estimator, we calculate its spectral decomposition and set all eigenvalues smaller than $4\gamma(\varepsilon)$ equal to zero, as prescribed in Supplementary Reference 1. After this thresholding procedure, the spectrum is no longer normalised, and we correct this by shifting all eigenvalues by the same quantity (as opposed to dividing by its sum). The average rank, over 100 experiments, obtained by this method is shown in Supplementary Figure 9. It is clear that the method performs well, as expected, for the informationally complete case. However, when not enough measurements are used in the reconstruction, it seems to (on average) estimate a rank that is greater than what our thresholding method gives (see Supplementary Figure 8). In terms of risk, as defined in eq. (1), and as shown in Supplementary Figure 11, we cannot see a very clear trend that distinguishes both methods, except that on average they seem to be similar in risk. Furthermore, we should notice that for the informationally incomplete regime we are interested in analysing, to date there is not a rigorously proven method for spectral thresholding available.

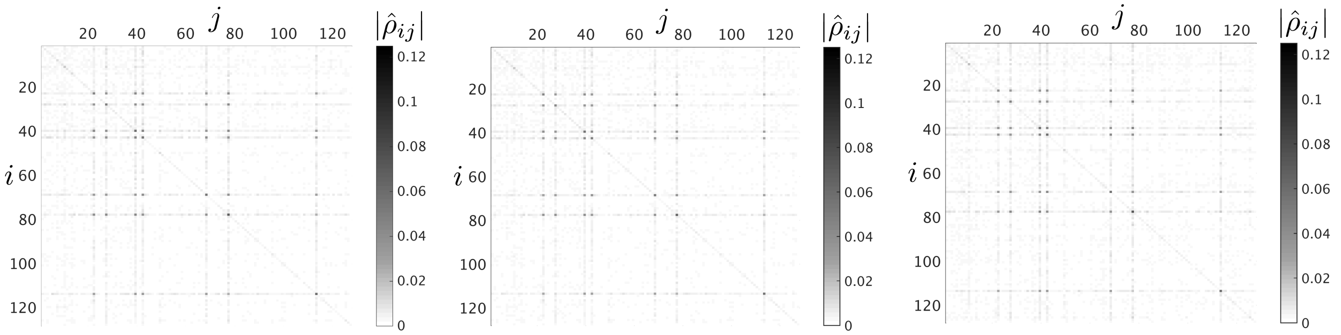

(a) (b) (c)

Supplementary Figure 1. Example of quantum state reconstruction for the logical $|\bar{1}\rangle$ state vector. A 2-D plot of the absolute values of the entries of the density matrix is presented. The same notation as in Fig. 1 in the main text is used. (a) Trace norm minimiser (TNM) estimate with minimal error level $\epsilon = 1.4$ ($F = 0.26$), corresponding to eq. (8) of the main text. (b) Least squares (LS) estimate ($F = 0.23$), corresponding to eq. (9) of the main text. (c) Rank 37 leading subspace projection of the least squares estimate ($F = 0.25$) obtained by our spectral thresholding method, eq. (18) of the main text.

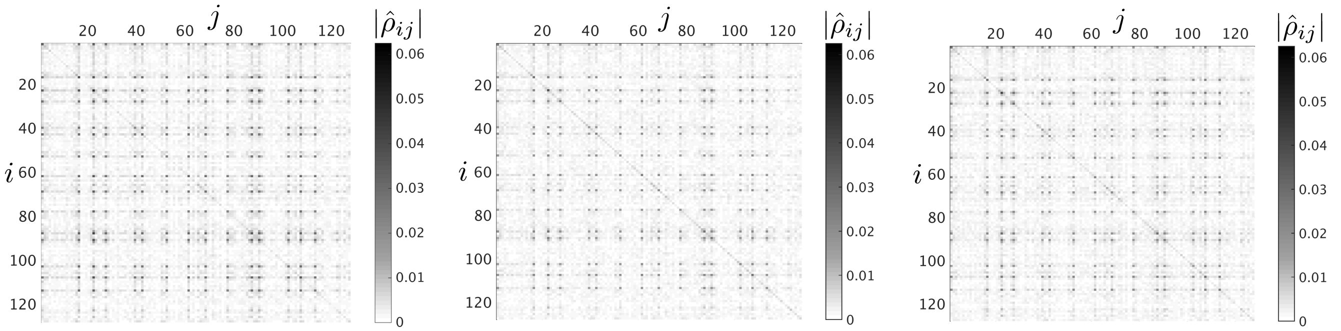

(a) (b) (c)

Supplementary Figure 2. Example of quantum state reconstruction for the logical $(|\bar{0}\rangle + |\bar{1}\rangle)/\sqrt{2}$ state vector. A 2-D plot of the absolute values of the entries of the density matrix is presented. The same notation as in Fig. 1 in the main text is used. (a) Trace norm minimiser (TNM) estimate with minimal error level $\epsilon = 1.5$ ($F = 0.44$), corresponding to eq. (8) of the main text. (b) Least squares (LS) estimate ($F = 0.29$), corresponding to eq. (9) of the main text. (c) Rank 41 leading subspace projection of the least squares estimate ($F = 0.32$) obtained by our spectral thresholding method, eq. (18) of the main text.

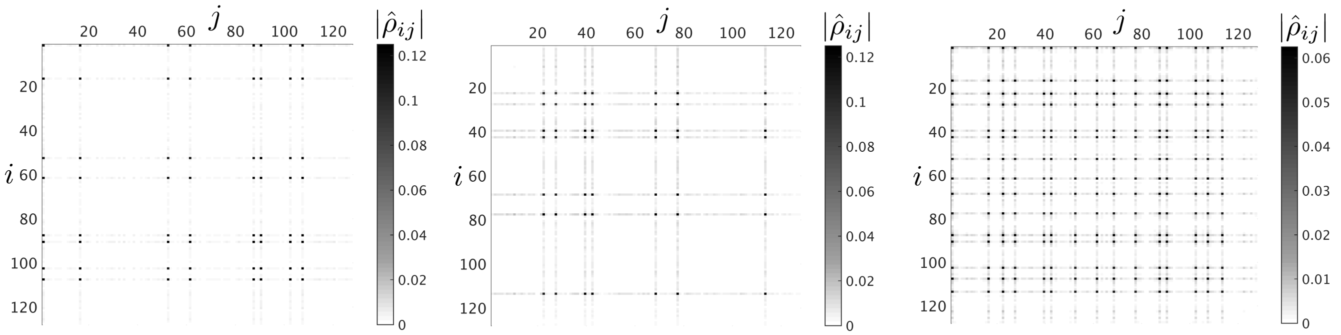

(a) (b) (c)

Supplementary Figure 3. Example of quantum state reconstruction via trace norm minimization for a large error parameter ($\epsilon = 1.8$) for the three logical states produced in our experiment. A 2-D plot of the absolute values of the entries of the density matrix is presented. The same notation as in Fig. 1 in the main text is used. (a) Logical $|\bar{0}\rangle$ state ($F = 0.98$). (b) Logical $|\bar{1}\rangle$ state ($F = 0.91$). (c) Logical $(|\bar{0}\rangle + |\bar{1}\rangle)/\sqrt{2}$ state ($F = 0.94$). Note that since the TNM estimator is used, the purity of the reconstructed states strongly depends on the value of the error parameter ϵ.

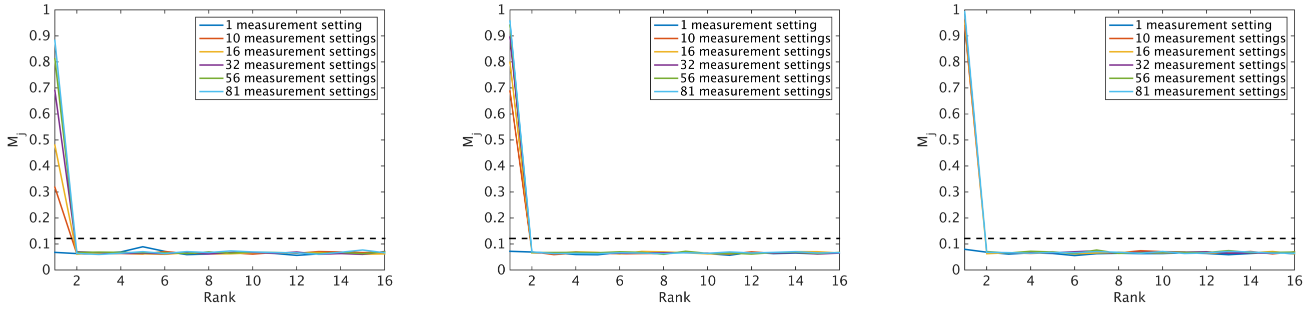

(a) (b) (c)

Supplementary Figure 4. Mean eigenvector overlap as a function of the rank truncation for the recovered density matrix of a true state of rank 1. (a) 5 repetitions per measurement setting. (b) 16 repetitions per measurement setting. (c) 100 repetitions per measurement setting. The dotted line represents the threshold, eq. (17) of the main text.

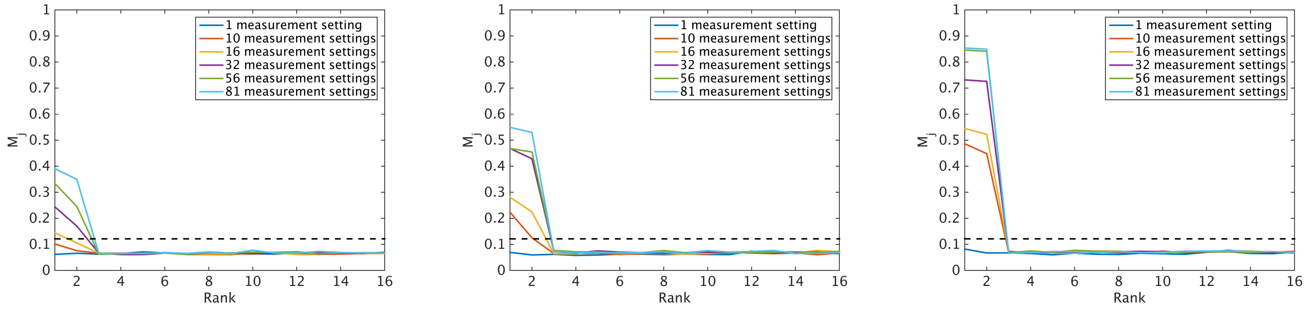

(a) (b) (c)

Supplementary Figure 5. Mean eigenvector overlap as a function of the rank truncation for the recovered density matrix of a true state of rank 2. (a) 5 repetitions per measurement setting. (b) 16 repetitions per measurement setting. (c) 100 repetitions per measurement setting. The dotted line represents the threshold, eq. (17) of the main text.

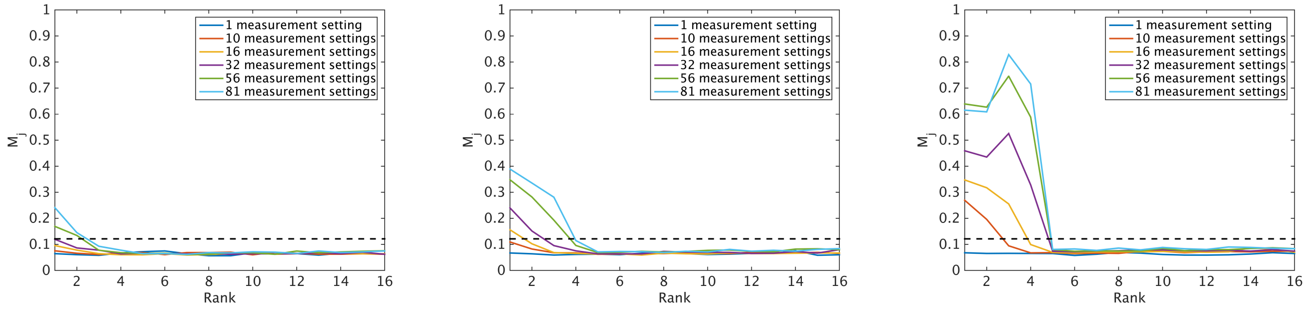

(a) (b) (c)

Supplementary Figure 6. Mean eigenvector overlap as a function of the rank truncation for the recovered density matrix of a true state of rank 4. (a) 5 repetitions per measurement setting. (b) 16 repetitions per measurement setting. (c) 100 repetitions per measurement setting. The dotted line represents the threshold, eq. (17) of the main text.

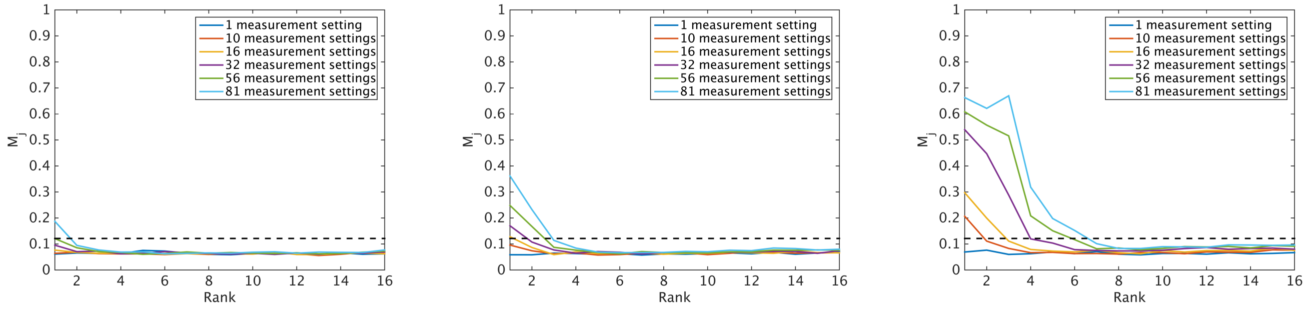

(a) (b) (c)

Supplementary Figure 7. Mean eigenvector overlap as a function of the rank truncation for the recovered density matrix of a true state of rank 8. (a) 5 repetitions per measurement setting. (b) 16 repetitions per measurement setting. (c) 100 repetitions per measurement setting. The dotted line represents the threshold, eq. (17) of the main text.

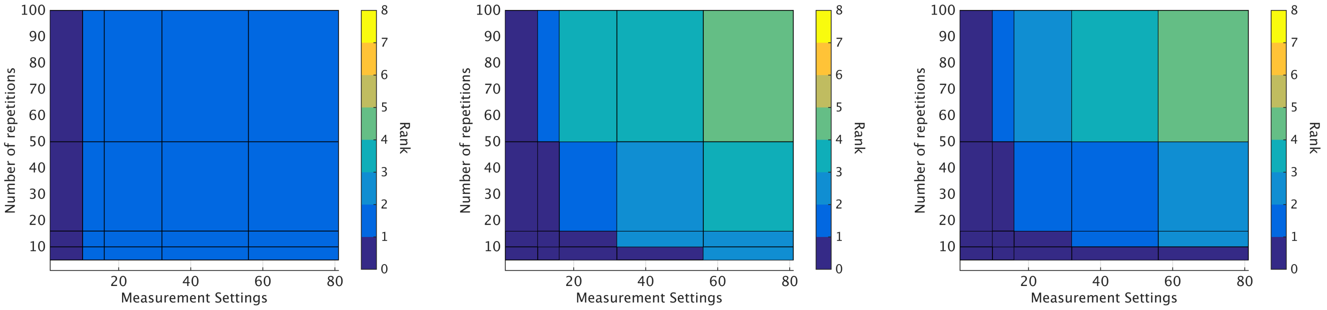

(a) (b) (c)

Supplementary Figure 8. Estimated rank according to spectral thresholding as a function of the number of measurement settings and number of repetitions per measurement. The color scales denotes the estimated rank. (a) Rank 1 true state. (b) Rank 4 true state. (c) Rank 8 true state. When insufficient information is available for our criterion to provide a non-zero rank, we choose a rank one estimate by default.

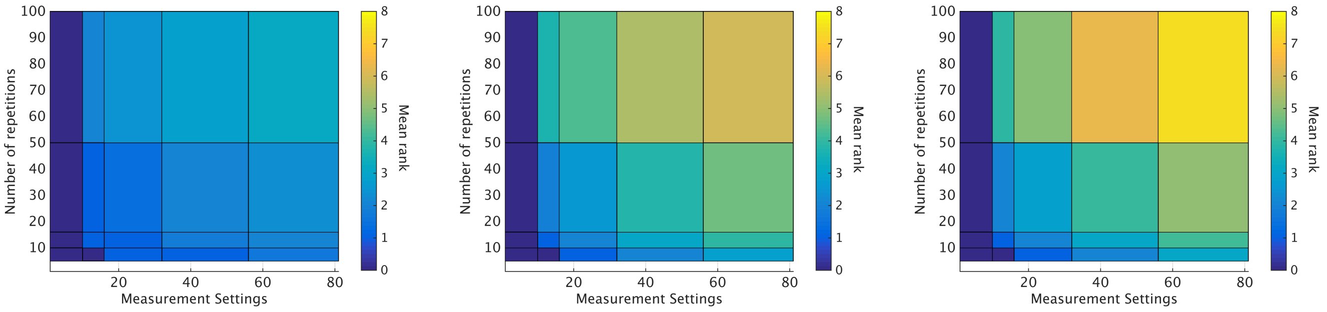

(a) (b) (c)

Supplementary Figure 9. Estimated mean rank according to the spectral thresholding method developed in Supplementary Reference 1 with positivity constraint. (a) Rank 1 true state. (b) Rank 4 true state. (c) Rank 8 true state. The method seems to overfit a little when not enough measurements are used in the reconstruction. Also, note that we are plotting the mean reconstructed rank, which does not take discrete values.

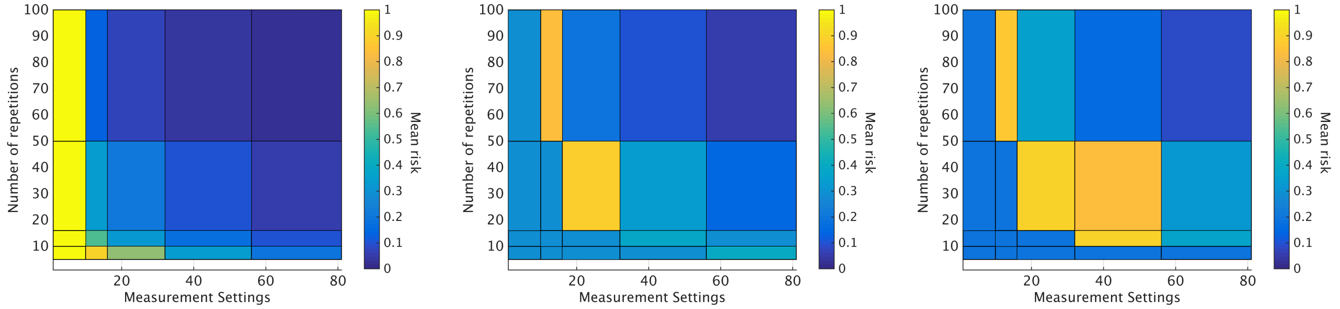

(a) (b) (c)

Supplementary Figure 10. Mean risk, eq. (1), computed for our thresholding procedure. (a) Rank 1 true state. (b) Rank 4 true state. (c) Rank 8 true state.

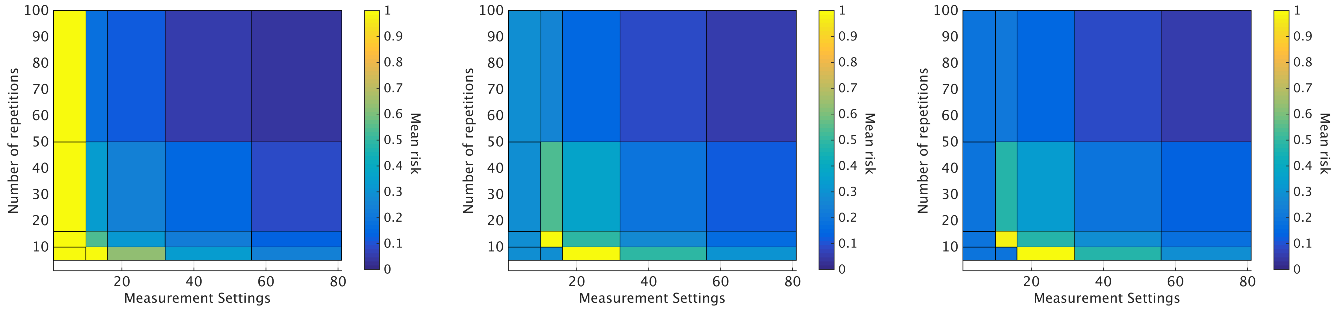

(a) Rank 1 true state. (b) Rank 4 true state. (c) Rank 8 true state.

Supplementary Figure 11. Mean risk, eq. (1), computed for the thresholding procedure in Supplementary Reference 1 with positivity constraint.

SUPPLEMENTARY REFERENCES

[1] C. Butucea, M. Guta, and T. Kypraios, (2015), arXiv:1504.08295.
